# DNA Barcoding and Phylogenetic Relationship of *Parabuthus liosoma* (Ehrenberg, 1828) (Scorpiones: Buthidae) in Saudi Arabia

**DOI:** 10.3390/biology15040321

**Published:** 2026-02-12

**Authors:** Ahmed Badry, Abdulmani H. Al-Qahtni, Abdullah M. Al-Salem, Manal S. Al Balawi, Fahad Mesfer, Wasayf S. Allahyani, Abdulaziz R. Alqahtani

**Affiliations:** 1 Department of Invertebrates, National Center for Wildlife, Riyadh 12411, Saudi Arabia; abdumani@ncw.gov.sa (A.H.A.-Q.); a.alsalem@ncw.gov.sa (A.M.A.-S.); m.albalawi@ncw.gov.sa (M.S.A.B.); f.almuflah@ncw.gov.sa (F.M.); wasayf@ncw.gov.sa (W.S.A.); 2 Department of Biology, College of Science, University of Bisha, P.O. Box 551, Bisha 61922, Saudi Arabia; arabe@ub.edu.sa

**Keywords:** molecular phylogeny, Scorpiones, mitochondrial DNA

## Abstract

(1) Background. *Parabuthus liosoma* is one of the largest buthid scorpion species and is endemic to Saudi Arabia and Yemen. This study provides the first DNA barcoding and phylogenetic analysis of *P. liosoma* from Saudi Arabia, contributing to global efforts in arachnid molecular identification and biodiversity documentation. (2) Methods. The whole genome was extracted from nine adult individuals of *P*. *liosoma,* collected from Farasan Island, southwest of Saudi Arabia. A portion of the mitochondrial DNA, specifically, the cytochrome oxidase subunit I gene (COI) sequences, was amplified and sequenced and subjected to genetic and phylogenetic analyses. (3) Results. The DNA barcoding results revealed a high level of genetic variability within *P. liosoma*, aiding in species identification and supporting its utility as a molecular tool for scorpion taxonomy. In addition, our results reveal a monophyletic relationship among *Parabuthus* species, with a clear distinction between Arabian and African lineages. (4) Conclusions. This study highlights the effectiveness of DNA barcoding as a reliable tool for species identification and taxonomy and enhances our knowledge of the evolutionary history and geographic distribution of *Parabuthus* scorpions. However, further research is required to elucidate the complex phylogenetic relationships within this genus.

## Simple Summary

Accurate species identification is essential for effective conservation of biodiversity. *Parabuthus liosoma* is one of the largest buthid scorpion species, which is restricted to Saudi Arabia and Yemen. For the first time in Saudi Arabia, DNA barcoding is used as a tool to enhance our knowledge of the evolutionary history of this species. DNA barcoding reveals high genetic diversity in *P. liosoma*, aiding species identification and validating its use in scorpion taxonomy. Also, *Parabuthus* shows a monophyletic relationship with distinct Arabian and African lineages, highlighting biogeographic divergence.

## 1. Introduction

Arthropods represent one of the most dominant and diverse animal groups on Earth. With this phylum, Arachnida exhibits a significant taxonomic diversity [1,2,3]. Scorpiones, a notable order of arachnids, have achieved remarkable evolutionary success among invertebrates, with over 2870 described species, due to their adaptability and wide distribution across various habitats [4]. The family Buthidae constitutes a major lineage within Scorpiones, with 94 genera and 1276 species [4]. Notably, this family is known for its medical importance due to the highly potent neurotoxins found in all of its species [5]. These toxins are specific to mammals, making them a potential threat to human health.

The genus *Parabuthus* was introduced by Pocock in 1890, with 42 described species [4]. It is distributed throughout the Afrotropical region, including Africa and Arabia [6,7]. This genus is known for various reasons, such as being the largest buthids and the only diurnal scorpion within this group, such as the *Parabuthus villosus* [8]. Stridulatory organs are located in all *Parabuthus* species, which produce an audible sound when the scorpion feels threatened [9]. Additionally, *Parabuthus* species are fossorial and burrow into specialized substrata [7]. This burrowing behavior, coupled with specialized ecomorphological adaptations, contributes to their remarkable diversity and abundance in arid regions [10]. The genus *Parabuthus* includes some of the world’s most venomous scorpions, which are responsible for many serious scorpion envenomations in southern Africa [11]. The distinct venom compositions of these species likely account for the variations in clinical effects and severity observed in scorpionism cases [12]. Kovařík et al. [13] conducted the first taxonomic review for all *Parabuthus* scorpion species in the Horn of Africa. Their in-depth study, based on a large collection of specimens, revealed that the previously recognized *P*. *liosoma* complex actually comprises three distinct, closely related species. These sibling species, each with its own geographic range, are now identified as *P*. *abyssinicus* Pocock, 1901 inhabits Eritrea, Djibouti, and northeastern parts of Ethiopia; *P*. *liosoma* (Ehrenberg, 1828), which inhabits Yemen and Saudi Arabia; and *P*. *maximus* Werner, 1913 inhabits Tanzania and Kenya.

Molecular phylogenetic studies are effective tools for assessing genetic divergence within and among populations, enhancing our understanding of scorpion diversification. [14] published the first molecular phylogeny for 17 genera of the Buthidae family using 16S rRNA mitochondrial markers. The diversification of scorpions has been greatly impacted by mountainous terrain and riverine barriers [15,16,17]. These studies emphasize how tectonic events, paleogeography, and paleobiogeography shape endemism and diversity in scorpions, especially in areas with high species richness and distinct evolutionary lineages [18,19,20,21,22]. Moreover, molecular phylogeny has been employed to interpret the evolutionary relationships among different populations and/or species of scorpions [23,24,25,26,27,28,29]. In addition, the COI gene serves as the gold-standard marker due to its hypervariable 5′ region, which generates distinct interspecific divergence while maintaining intraspecific stability. It enables reliable discrimination of closely related species and resolving cryptic diversity within scorpion genera such as *Androctonus*, *Buthus*, *Buthacus*, *Hottentotta*, *Leiurus*, *Parabuthus*, and *Scorpio* [30,31,32,33,34].

The aim of this work is to construct a reference database for *P*. *liosoma* using COI-based barcoding. Also, to document and evaluate the phylogenetic relationship among *P*. *liosoma* from Saudi Arabia and the available comparative sequence data of this genus in the GenBank, based on the COI gene.

## 2. Materials and Methods

### 2.1. Scorpion Collection

A total of nine adult specimens of *P*. *liosoma* were collected from Farasan Island, southwest of Saudi Arabia, during a field survey conducted from 27 February 2023 to 3 March 2023 (Table 1). The specimens were collected mainly at night using ultraviolet light techniques and randomly searching under rocks or other potential shelters during the daytime [35]. The scorpions were subsequently preserved in 95% ethanol as described by [36].

### 2.2. Molecular Analysis

Genomic DNA was extracted and collected from leg tissue of preserved specimens using the commercially available DNeasy Blood and Tissue Kit (Qiagen, Hilden, Germany) following the manufacturer’s protocol. To assess DNA quality, 5 μL of genomic DNA was mixed with 1 μL of 6× DNA loading buffer (containing 0.25% w/v bromophenol blue and 40% w/v sucrose) and loaded onto a 1% agarose gel stained with 0.5 μg/mL ethidium bromide (Thermo Fisher Scientific, Waltham, MA, USA). A portion of the COI gene was amplified using standard polymerase chain reaction (PCR) technique. The amplification process employed with invertebrate universal primers LCO1490 (5′-GGTCAACAAATCATAAAGATATTGG-3′) and HCO2198 (5′-TAAACTTCAGGGTGACCAAAAAATCA-3′), as described by [39,40]. The PCR amplification consisted of (1) an initial denaturation at 94 °C for 5 min; (2) 30 cycles of denaturation at 94 °C for 30 s, annealing at 48 °C for 30 s, and extension at 72 °C for 30 s; and (3) a final extension at 72 °C for 7 min. Products of the PCR were electrophoresed in a 1% agarose gel stained with 0.5 μg/mL of ethidium bromide, observed by using UV transilluminator, and subsequently purified. The purified COI gene fragments were then sequenced using an ABI 3500 automated sequencer (Applied Biosystems, Foster City, CA, USA). The generated DNA sequences were deposited in GenBank (Table 1) at https://www.ncbi.nlm.nih.gov/genbank/, accessed on 25 April 2023 with accession numbers (PV124846-PV124854).

### 2.3. Phylogenetic Analysis

The obtained sequences were analyzed, screened, and filtered using FINCH TV 1.4.0 (Geospiza, Inc., Seattle, WA, USA; http://www.geospiza.com (accessed on 25 April 2023). Then, each sequence was individually queried and compared with the GenBank database using BLASTn tools (http://blast.ncbi.nlm.nih.gov/Blast.cgi (accessed on 25 April 2023)). To enhance our phylogenetic analysis, we incorporated additional comparative sequences for the genus *Parabuthus* and other related Buthidae taxa from GenBank. While the COI gene is represented by a substantial number of entries, providing robust comparative data. *Buthus mardochei* (ON255580.1) and *Microbuthus kristensenorum* (ON255621.1) were selected as an outgroup taxon in order to root the phylogenetic tree. Also, the DNA sequences were aligned using the CLUSTALW program with its default settings to analyze the genetic data [41]. Subsequently, we calculated the nucleotide composition for each group of related species, considering only the sequences within that specific group. Also, MEGA 11 software [41] was used to determine the genetic distances between all pairs of sequences within the entire dataset. In addition, the genetic distance matrix was visualized by a heatmap using the R heatmap function clustering package (http://www.r-project.org/ (accessed on 25 April 2023)). The maximum parsimony (MP) and neighbor-joining (NJ) analyses in PAUP v.4 were used to infer phylogenetic relationships based on the COI dataset (*n* = 34) [42]. To find the most efficient tree topologies based on TBR branch swapping, heuristic research was performed. These analyses did not include characters with missing data, with 1000 replicates to evaluate bootstrap support for nodes [43]. Mrmodeltest v. 2.3 [44] was also used to select the best nucleotide substitution model based on the Akaike Information Criterion (AIC) [45]. Moreover, Mrbayes v. 3.1.2 [46] was used to perform Bayesian inference analysis (BI) to infer biogeographic patterns and reconstruct phylogenetic relationships. For one million generations, the MCMC analysis was performed. Tracer v. 1.7 was used to visualize and summarize posterior probabilities [47].

## 3. Results

BLASTn comparisons revealed the absence of exact species-level matches for *Parabuthus liosoma* in GenBank (Table 2). The highest similarity was observed with congeners *Parabuthus kajibu* (90.91%), *P. brevimanus* (89.35%), and *P. pallidus* (89.16%), highlighting the lack of direct genomic data for *P. liosoma*. Unexpectedly, sequences from distantly related genera like *Centruroides granosus* (88.24%) and *Centruroides luceorum* (88.21%) showed moderate identity levels. This absence of 100% identity matches suggests either unresolved genetic divergence within the *Parabuthus* genus or the need for targeted sequencing of *P. liosoma* to refine its molecular characterization, underscoring taxonomic ambiguities and gaps in current genomic repositories.

### 3.1. Genetic Data

A total of 500 aligned nucleotides of the cytochrome oxidase I (COI) gene were analyzed in a group of *Parabuthus* scorpions. This region exhibited a high degree of variability, with approximately 36% of the nucleotide positions differing among individuals. The nucleotide composition showed a strong bias towards adenine (A) and thymine (T), which is a common characteristic of many invertebrate genomes. Analysis revealed 23 segregating sites where multiple DNA sequences were observed, indicating genetic diversity within the population.

### 3.2. Genetic Distance

**Figure 1:**
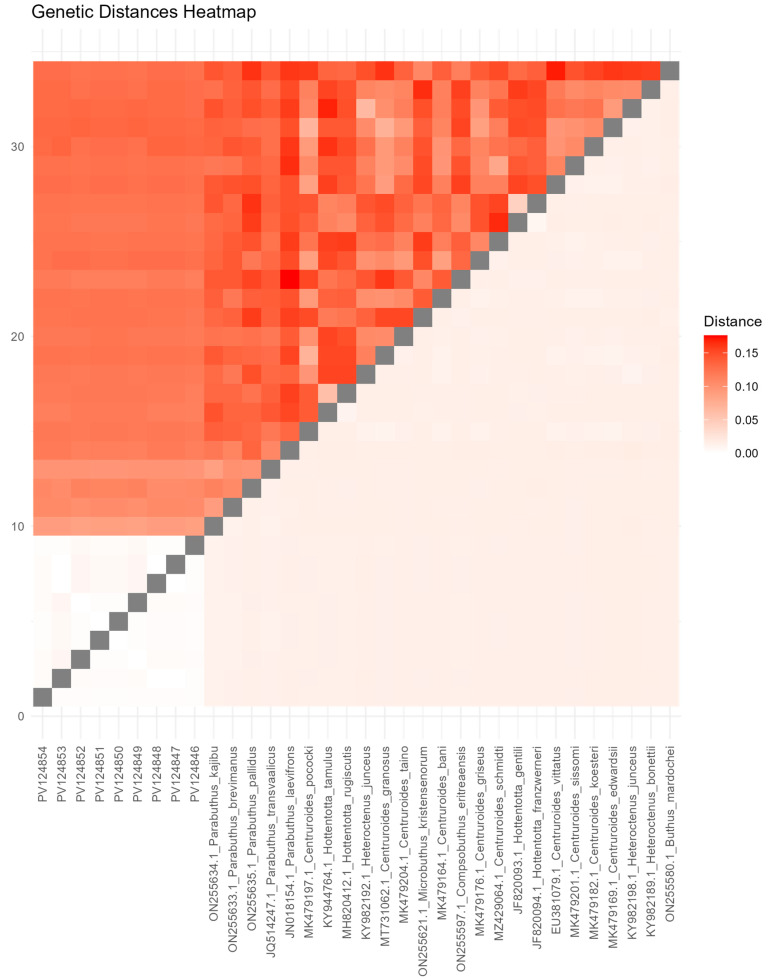
Pairwise genetic distance heatmap of *Parabuthus liosoma* with it congeners and other related Buthidae scorpion species based on COI data sequences.

The level of genetic divergence among the different *Parabuthus* taxa varied considerably, ranging from a very low 0.2% to a substantial 11.8%, with an average of 3.6% (Figure 1, Table S1). *P. liosoma* (PV124846) demonstrates low genetic relatedness to other *Parabuthus* samples present in the dataset (PV124854-PV124847). Comparatively, *P. liosoma* exhibits greater genetic distances to other members within the same genus, including *P. kajibu* (ON255634.1), *P. brevimanus* (ON255633.1), *P. pallidus* (ON255635.1), *P. transvaalicus* (JQ514247.1), and *P. laevifrons* (JN018154.1). While between other Buthidae genera, such as *Centruroides*, *Hottentotta*, and *Heteroctenus*, the genetic distance ranged between 11.6% and 13.2% (Figure 1, Table S1).

### 3.3. Phylogenetic Analyses

**Figure 2:**
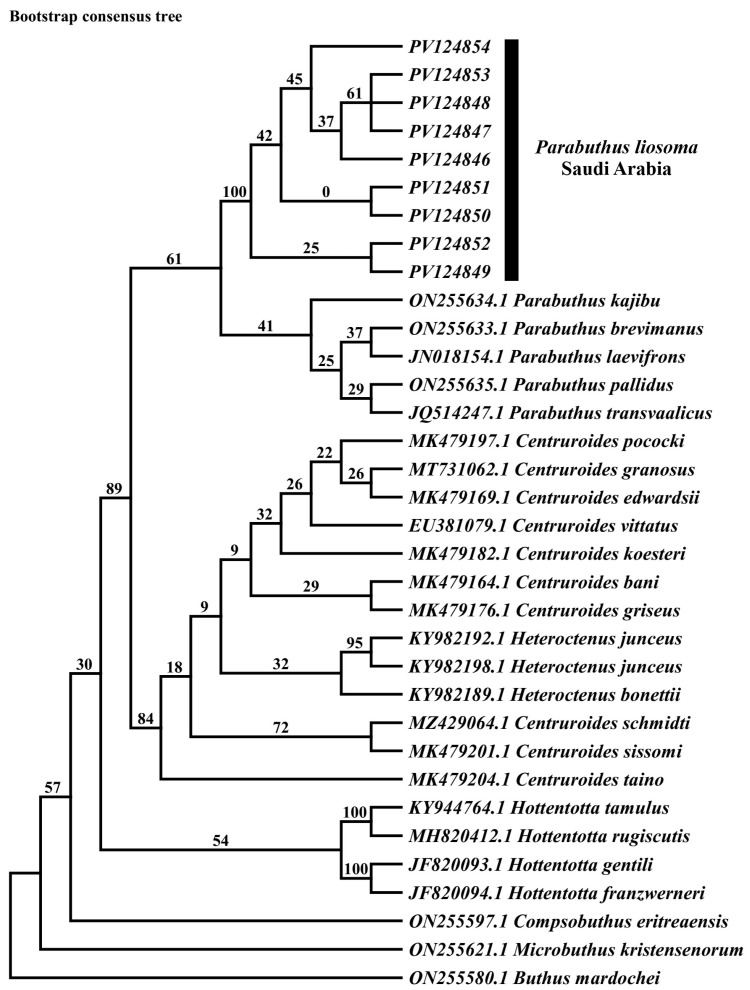
Maximum parsimony phylogenetic tree of genus *Parabuthus* and other Buthidae-related sequences of the COI gene. The numbers above branches indicate bootstrap values calculated with 1000 replicates.

**Figure 3:**
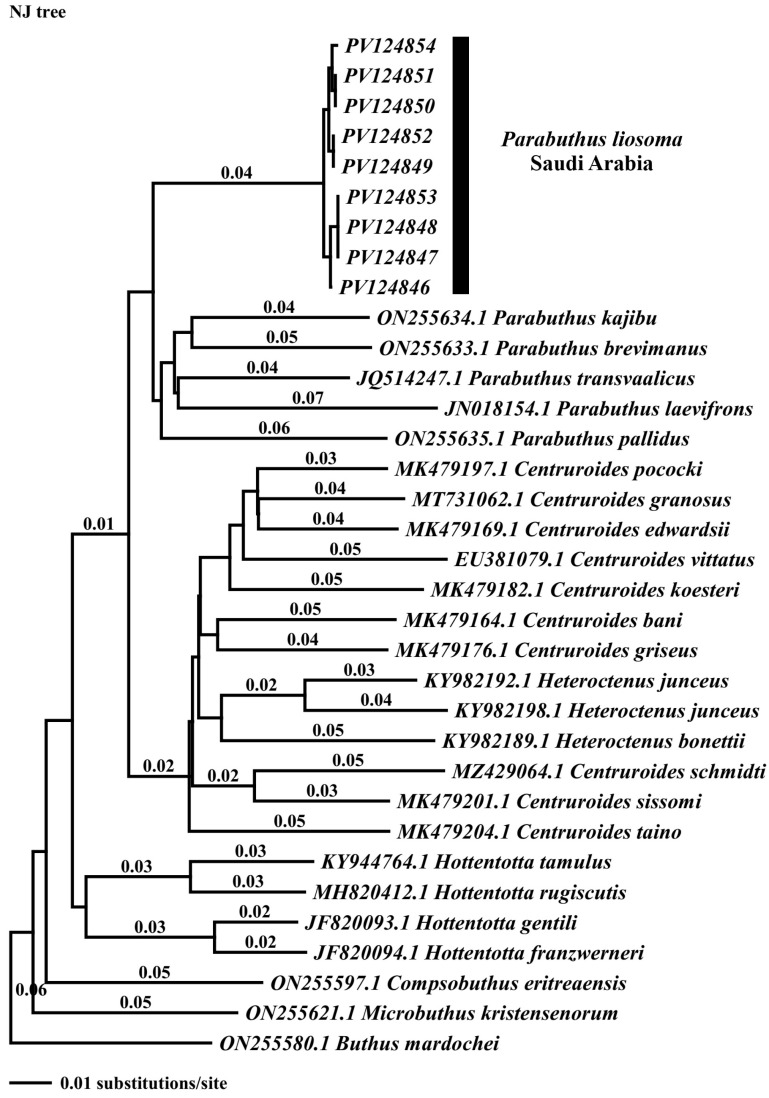
Neighbor-joining phylogenetic tree of genus *Parabuthus* and other Buthidae-related sequences of the COI gene. The numbers above branches indicate distance values.

**Figure 4:**
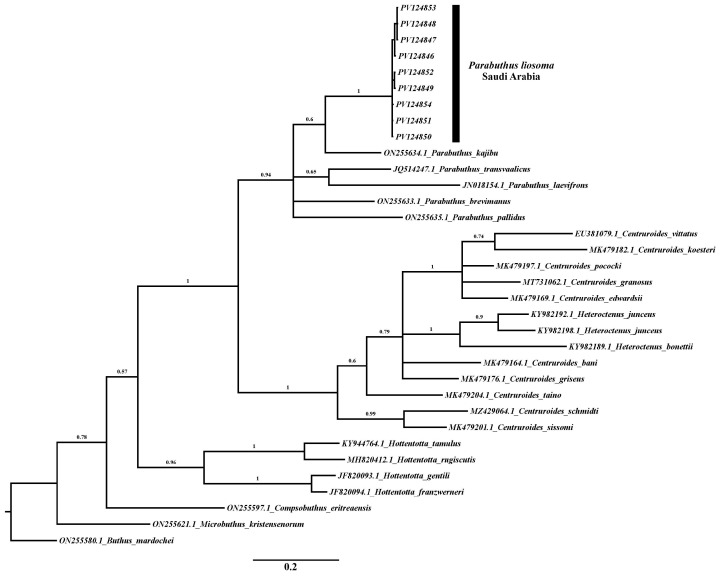
Bayesian inference phylogenetic tree of genus *Parabuthus* and other Buthidae-related sequences of the COI gene. The numbers above indicate the posterior probabilities.

We conducted the maximum parsimony analysis using sequences with 500 bp in length, treating gaps as missing data. which produced two most-parsimonious trees with a length of 625 steps (homoplasy index = 0.6611; consistency index = 0.3389; retention index = 0.5355). The topology resulting from maximum parsimony (MP), neighbor joining (NJ) tree, and Bayesian inference (PI) analysis was identical (Figure 2, Figure 3 and Figure 4). Two distinct and strongly supported clades were identified in all phylogenetic analyses, both represented by *Parabuthus* taxa. The first clade consisted of nine specimens of *P. liosoma* (PV124854-PV124847) from Farasan Island, Saudi Arabia (bootstrap = 100%; posterior probability = 1; Figure 3 and Figure 4). The second clade included the remaining sequences retrieved from GenBank belonging to the genus *Parabuthus*, grouped with a sister relationship with the remaining clades encompassing all related *Buthidae* taxa (Figure 2, Figure 3 and Figure 4).

## 4. Discussion

Scorpions in Saudi Arabia pose a significant public health concern due to the local desert terrain, warm climate, and their venomous effects, with approximately 14,500 scorpion stings per year [48,49]. Also, scorpion taxonomy and identification have traditionally relied on morphological traits, such as body segmentation, pedipalp characteristics, and trichobothria patterns. However, this approach is often limited by cryptic species and morphological similarities that can lead to taxonomic misidentifications or the inability to identify species [50]. DNA barcoding based on COI has emerged as a robust alternative for species identification and provides a standardized sequence that is highly conserved across species to generate a barcode gap to infer species boundaries [31,50]. In addition, DNA barcoding remains an essential tool for resolving taxonomic ambiguities and improving the accuracy of scorpion identification [33]. *P. liosoma* subjected to DNA barcoding in the current study and described morphologically in previous research is one of the few representative scorpion species that represent a significant hazard for human health [48]. In our data, the level of COI divergence among *P. liosoma* in Saudi Arabia and the other congeneric species was only about ten times higher than intraspecific genetic distance (Figure 1, Table S1). Our finding aligns with the conclusion of [51], who proposed a distance-based approach suggesting that interspecific genetic divergence should typically be about ten times higher than intraspecific divergence.

Our phylogenetic analyses consistently indicate that the *Parabuthus* genus is monophyletic (Figure 3 and Figure 4) based on the COI gene. Previous assemblage studies based on molecular, morphological, and behavioral characters suggested the monophyly of the genus *Parabuthus* to be likely to form a single evolutionary lineage [7,37,52]. However, more extensive studies are needed, including a broader sampling of *Parabuthus* species, particularly from undersampled regions, to further refine the classification and fully resolve the phylogenetic relationships within this group. Further studies are required with additional molecular markers and comprehensive geographic sampling to know more about the evolutionary history and biogeographical patterns of *Parabuthus* scorpions.

Our phylogenetic analysis of the Saudi Arabian *P. liosoma* sequences being basal to these other species of the same genus, including *P. kajibu*, *P. brevimanus*, *P. laevifrons*, *P. pallidus*, and *P. transvaalicus*. Also, *Parabuthus* species reveals a distinct geographic separation or splitting between the *P. liosoma* samples on Farasan Island in Saudi Arabia and those found in southern and northeastern Africa. This splitting is supported by a genetic divergence of 9–11% (Table S1). Notably, *P*. *liosoma* can be differentiated from the above species based on smooth granulation of the pedipalp patella, as described by [13]. It is worth noting that the geographical range of *P. liosoma* is restricted to Yemen and Saudi Arabia, according to [13]. Several authors also referred to the presence of vicariance events associated with the formation of the Red Sea, which led to simultaneous differences between Arabian and African lineages [53,54]. Notably, *P. abyssinicus*, historically considered a synonym of *P. liosoma*, was revalidated as a distinct species within a species complex by [13]. However, [55,56,57] recorded this species (*P*. *liosoma*) from the Elba Protected Area, Red Sea, Egypt. Thus, further morphological investigations with additional specimens may reveal possible undiscovered species in this region. Prendini et al. [36] proposed that the burrowing scorpion originated in Africa and subsequently dispersed into other regions of Africa during the Early Miocene. This hypothesis aligns with numerous molecular phylogenetic and biogeographic studies, which suggest that the Arabian Plate facilitated the dispersal of these scorpions during the Miocene epoch [58,59]. Also, geological events, such as continental drift, mountain building, and the formation of land bridges, combined with climatic-vegetational fluctuations, have been identified as major factors influencing present-day scorpion distributions [60,61]. Further studies are critically required to evaluate the taxonomic status and phylogenetic relationship based on multilocus genes, specifically in scorpion species native to Saudi Arabia.

## 5. Conclusions

This study provides the first DNA barcode records for the medically important scorpion species *Parabuthus liosoma* in Saudi Arabia. The generated barcodes are essential for standardizing global reference databases, particularly given existing taxonomic inconsistencies. Also, the phylogenetic analyses support the monophyly of the *Parabuthus* genus and suggest a significant genetic distinction between the Arabian populations of *P. liosoma* samples from Farasan Island, Saudi Arabia, and those from southern and northeastern African species based on the COI gene. The study also revealed that *Parabuthus* are sister taxa, consistent with previous findings. The results provide insights into evolutionary history and geographic distribution and highlight the need for further research with additional representative species of the *Parabuthus* genus.

## Figures and Tables

**Table 1:** Localities, sex, and GenBank accession numbers for *Parabuthus liosoma* specimens collected from Saudi Arabia used in this study.

No.	Country	Species	N	E	Sex	Accession No.	Authority
1	Saudi Arabia	*Parabuthus liosoma*	16.9608	41.9269	Male	PV124846	This study
2		*Parabuthus liosoma*	16.9608	41.9269	Female	PV124847	This study
3		*Parabuthus liosoma*	16.8640	41.9441	Male	PV124848	This study
4		*Parabuthus liosoma*	16.8640	41.9441	Female	PV124849	This study
5		*Parabuthus liosoma*	16.7620	41.9957	Male	PV124850	This study
6		*Parabuthus liosoma*	16.7620	41.9957	Male	PV124851	This study
7		*Parabuthus liosoma*	16.7556	42.0803	Female	PV124852	This study
8		*Parabuthus liosoma*	16.7556	42.0803	Juvenile	PV124853	This study
9		*Parabuthus liosoma*	16.6928	42.1426	Juvenile	PV124854	This study
10	*-*	*Parabuthus kajibu*	-	-		ON255634.1	[37]
11	*-*	*Parabuthus brevimanus*	-	-		ON255633.1	[37]
12	*-*	*Parabuthus pallidus*	-	-		ON255635.1	[37]
13	*-*	*Parabuthus laevifrons*	-	-		JN018154.1	[38]

**Table 2:** GenBank accession numbers of *Parabuthus liosoma* sequences from the isolates associated with blast and brown spot lesion and the similarity scores obtained by BLASTn search.

Submission	GenBank Accession No.	Similarity (%) to Existing Sequences
*Parabuthus kajibu*	ON255634.1	90.91
*Parabuthus brevimanus*	ON255633.1	89.35
*Parabuthus pallidus*	ON255635.1	89.16
*Centruroides bani*	OR894651.1	88.58
*Parabuthus transvaalicus*	JQ514247.1	87.85
*Centruroides luceorum*	MK479185.1	88.21
*Centruroides granosus*	MT731071.1	88.24
*Centruroides insulanus*	MK479180.1	88.03
*Hottentotta tamulus*	KY944760.1	87.85
*Centruroides pococki*	MK479197.1	87.89
*Parabuthus laevifrons*	JN018154.1	87.80

## Data Availability

Data are contained within the article.

## Supplementary Materials

The following supporting information can be downloaded at https://www.mdpi.com/article/10.3390/biology15040321/s1: Table S1: The uncorrected p distance of the sequence divergence of COI mtDNA sequences between *Parabuthus* samples and other related Buthidae species was included in this study.
